# Vibrational spectroscopy and multiphoton microscopy for label-free visualization of nervous system degeneration and regeneration

**DOI:** 10.1007/s12551-023-01158-2

**Published:** 2023-10-05

**Authors:** Roberta Galli, Ortrud Uckermann

**Affiliations:** 1 Medical Physics and Biomedical Engineering, Faculty of Medicine, Technische Universität Dresden, Dresden, Germany; 2 Department of Neurosurgery, Faculty of Medicine and University Hospital Carl Gustav Carus, Technische Universität Dresden, Dresden, Germany; 3 Division of Medical Biology, Department of Psychiatry and Psychotherapy, Faculty of Medicine and University Hospital Carl Gustav Carus, Technische Universität Dresden, Dresden, Germany

**Keywords:** Central nervous system, Nerve, Injury, Nonlinear microscopy, Label-free, Multiphoton

## Abstract

Neurological disorders, including spinal cord injury, peripheral nerve injury, traumatic brain injury, and neurodegenerative diseases, pose significant challenges in terms of diagnosis, treatment, and understanding the underlying pathophysiological processes. Label-free multiphoton microscopy techniques, such as coherent Raman scattering, two-photon excited autofluorescence, and second and third harmonic generation microscopy, have emerged as powerful tools for visualizing nervous tissue with high resolution and without the need for exogenous labels. Coherent Raman scattering processes as well as third harmonic generation enable label-free visualization of myelin sheaths, while their combination with two-photon excited autofluorescence and second harmonic generation allows for a more comprehensive tissue visualization. They have shown promise in assessing the efficacy of therapeutic interventions and may have future applications in clinical diagnostics. In addition to multiphoton microscopy, vibrational spectroscopy methods such as infrared and Raman spectroscopy offer insights into the molecular signatures of injured nervous tissues and hold potential as diagnostic markers. This review summarizes the application of these label-free optical techniques in preclinical models and illustrates their potential in the diagnosis and treatment of neurological disorders with a special focus on injury, degeneration, and regeneration. Furthermore, it addresses current advancements and challenges for bridging the gap between research findings and their practical applications in a clinical setting.

## Introduction

The central nervous system (CNS) and the peripheral nervous system (PNS) can be affected by traumatic and degenerative diseases, which often result in chronic neurological deficits and morbidity. While therapeutic approaches are currently being investigated, healing treatments are not always available for the PNS and are not yet possible in the CNS. Therefore, techniques that aid in understanding the mechanisms of nervous damage and characterizing therapeutic effects are highly valuable.

This review article focuses on vibrational spectroscopy and label-free multiphoton microscopy techniques for rapid and label-free histological and molecular characterization of the CNS and PNS in various pathological states. Vibrational spectroscopy encompasses two analytical techniques – infrared (IR) and Raman spectroscopy – which investigate molecular vibrations and provide spectra containing information about the overall biochemical composition. Label-free multiphoton microscopy allows for the simultaneous acquisition of both detailed structural and chemical information within biological samples. While commonly used in biomedical research for exciting and analyzing fluorescent dyes (Sanderson 2023), multiphoton microscopy can provide chemical selectivity by exploiting the simultaneous acquisition of multiple nonlinear signals originating directly from endogenous tissue constituents.

Label-free tissue imaging offers distinct advantages. First, it eliminates the need for sample sectioning. Unlike light and fluorescence microscopy, label-free microscopy does not require histological or immunohistochemical staining of tissues. This minimizes the potential for staining-related artifacts and further preserves the natural state of the sample. Immunohistochemistry, while powerful, can yield variable results due to multiple preparation factors. Label-free imaging offers improved reproducibility, making it easier to compare and replicate results across different studies. In the context of nervous tissue, this is particularly valuable for quantitative measurements like axon morphometry, which otherwise exhibits high variability depending on experimental protocols (Saliani et al. 2017).

Toward monitoring the effects of experimental regenerative and neuroprotective therapies, spectroscopy provides information on biochemical changes within the injured tissue, while label-free microscopy allows for a more comprehensive visualization of degenerating and regenerating nervous structures, axons, and cells. Importantly, label-free methods are predestinated for in vivo tissue analysis. Stained tissue sections used in conventional microscopy limit the number of endpoints available for studying disease progression or therapy effects in preclinical studies. In vivo imaging provides better time resolution, reducing the use of laboratory animals. Moreover, label-free microscopy avoids potential perturbations associated with dye intercalation, non-specific binding, compartmentalization, leakage, and spectral overlap, which can occur in conventional intravital microscopy based on fluorescence-based methods (Pittet and Weissleder 2011).

Therefore, label-free in vivo microscopy can reveal intricate processes that are challenging to elucidate with conventional histological methods. This opens up new avenues for studying axonal regeneration and the effects of innovative therapeutic approaches (Zheng et al. 2019). In the long run, label-free microscopy may facilitate the translation of in vivo histopathology into a diagnostic tool in the clinical context.

## Techniques and instrumentation for label-free imaging and analysis of myelin-rich structures, cells, and extracellular matrix of the nervous tissue

Optical methods provide the opportunity for label-free tissue characterization by utilizing various processes of light interaction with endogenous tissue molecules. Several techniques have been employed to investigate the structure and composition of nervous tissue, namely: Fourier-transform infrared (FT-IR) and Raman spectroscopy, coherent Raman scattering microscopy (including stimulated Raman scattering (SRS) and coherent anti-Stokes Raman scattering (CARS)), second and third harmonic generation (SHG, THG), as well as two-photon excited autofluorescence (TPEF) microscopy. Main characteristics and applications including already realized in vivo translation are summarized in Table 1.

**Table 1:** Methods of vibrational spectroscopy and label-free multiphoton microscopy used for CNS and PNS studies with key publications for in vivo and endoscopic applications

Methods		Excitation	Output	Biological targets in the nervous tissue	Experimental applications in vivo	Endoscopy
Linear	FT-IR spectroscopy	Broadband MIR light	Spectrum; multimodal imaging by uni- or multivariate chemometrics	Molecular vibrations, whole biochemistry	None	Not used in this context
	Raman spectroscopy	VIS or NIR cw laser			Wu et al. 2022	Not used in this context
Nonlinear	CARS	Two NIR short-pulsed laser	Signal intensity; multimodal imaging by simultaneous acquisition of more signals at different wavelengths	Lipids (myelin)	Huff and Cheng 2007 (CARS + SHG) Bélanger et al. 2012 (CARS + TPEF)	Légaré et al. 2006
	SRS			Lipids (myelin), proteins		Saar et al. 2011
	TPEF	One NIR short-pulsed laser		Cellular and extracellular fluorophores: NAD(P)H, lipofuscin, elastin		Ducourthial et al. 2015 (multimodal imaging)
	SHG			Fibrillar collagens, tubulin		
	THG			Lipid/water interfaces (Cell membranes)		Kuzmin et al. 2016

### Spectroscopy

Infrared and Raman spectroscopy, as well as coherent Raman scattering, are all methods that explore molecular vibrations through linear and nonlinear interactions between light and matter (Geraldes 2020). However, each technique possesses its own advantages and limitations, making them non-equivalent approaches. FT-IR and Raman spectra provide valuable information regarding lipid content, type, and saturation degree, as well as the presence and quantity of carbohydrates, nucleic acids, and proteins (Barth and Zscherp 2002; Movasaghi et al. 2008; Talari et al. 2015). FT-IR absorption spectroscopy is particularly sensitive to probing the antisymmetric vibrations of polar groups, such as in proteins, while Raman spectroscopy excels in probing the symmetric vibrations of non-polar groups (Larkin 2018), such as in lipids (Czamara et al. 2015).

However, FT-IR spectroscopy is suited for thin samples like tissue cryosections and is limited by low spatial resolution due to mid-infrared excitation. Additionally, its application on hydrated biological material is hindered by the strong absorption of water (Uckermann et al. 2014). On the other hand, Raman spectroscopy, which is a reflection technique, can be performed on bulk samples and is technically compatible with both in vitro and in vivo measurements (Cordero et al. 2018; Downes and Elfick 2010). The use of visible or near-infrared (NIR) laser beams for excitation enables high spatial resolution. It is also insensitive to water. Nevertheless, the low yield significantly restricts its application for Raman mapping in living systems, as NIR Raman spectroscopy typically requires long acquisition times (Barton et al. 2019).

Spectroscopy offers the possibility to extract a comprehensive chemical information, allowing the exploration of multiple chemical parameters, a concept referred to as “spectromics” (Petibois 2017). The compositional “fingerprint” provides the capability to differentiate between tissue types through the application of chemometrics (Galli et al. 2012). To achieve this objective, both unsupervised methods, such as clustering and principal component analysis, as well as supervised classification methods, are well suited. These techniques group spectra based on similarities, enabling the creation of multiple tissue maps that highlight the local biochemical changes of interest. Nonetheless, obtaining precise information regarding biomolecule content remains challenging since most IR and Raman bands are associated with molecular groups (e.g., amide, methyl, and methylene) rather than specific molecules.

### Label-free multiphoton microscopy

Coherent Raman scattering is utilized in various ways in multiphoton microscopy. Single-band CARS microscopy is specifically designed to target a particular mode of vibration in a molecular bond, making it ideal for lipid imaging (Freudiger et al. 2008) and thus predestinated for applications in the nervous tissue, whenever imaging of myelin (the lipid-rich substance insulating the axons of all vertebrates) is needed. SRS microscopy offers another approach to generate images based on vibrational contrast and enables hyperspectral imaging of both lipids (and therefore of myelin) and proteins through fast tuning of the laser source (Ozeki et al. 2012). THG microscopy takes advantage of the nonlinear signal generated by water-lipid and water-protein interfaces, providing label-free visualization of myelin sheaths surface, of intra- and extracellular membranes as well as of extracellular matrix (ECM) structures (Weigelin et al. 2016). Since nonlinear optical processes require high photon densities achievable only in the focal point of tightly focused ultrashort lasers, all multiphoton imaging modalities are inherently confocal (Dunn and Young 2006; Zipfel et al. 2003).

The combination of different nonlinear techniques for visualization of myelin, such as CARS, SRS, or THG, in conjunction with TPEF and SHG, allows for the simultaneous acquisition of co-localized information about nervous tissue at subcellular resolution, as elaborated in the following sections. TPEF is employed for imaging various cellular and extracellular endogenous fluorophores (König 2000). SHG microscopy enables the specific imaging of structural proteins lacking inversion symmetry, such as fibrillar collagen, myosin, and tubulin (Aghigh et al. 2023).

Label-free multiphoton microscopy is typically integrated into laser scanning microscopes equipped with NIR-emitting lasers, which can penetrate deeper into the tissue (Helmchen and Denk 2005). The imaging depth depends on tissue properties but generally does not exceed 100 μm in strongly myelinated nervous tissue such as peripheral nerves (Rishøj et al. 2022). Modern techniques (such as SWIR excitation or wavefront shaping) have the potential to increase the imaging depth of multiphoton microscopy up to about 1 mm (Hofer et al. 2020; Miller et al. 2017; Rowlands et al. 2019; Xiao et al. 2023). Particularly, three-photon excited fluorescence has shown remarkable potential for imaging deep brain structures, although its primary application has historically been in conjunction with fluorescent labels (Xiao et al. 2023).

In vivo imaging in animal models can be achieved by surgically exposing the region of interest or using implanted window chambers. For example, long-term in vivo imaging with Raman spectroscopy as well as multiphoton microscopy of rodent spinal cord can be achieved using an intervertebral window and biocompatible clearing methods that preserve window transparency over time (Wu et al. 2022). Endoscopic needles, employing graded-index lenses, enable access to deep tissue during in vivo imaging (Bélanger et al. 2012; Dilipkumar et al. 2019).

However, these approaches are limited to small animal models that fit the microscope stage. To expand the application to large animal models or future clinical assessments of nerve regeneration, the development of multiphoton endoscopic systems is necessary. Fiber-based flexible endoscopes, integrating miniaturized lenses for focalization and standard single-mode glass fibers for laser excitation in the picosecond regime, were initially demonstrated for CARS imaging in 2006 (Légaré et al. 2006). Subsequently, endoscopic systems capable of generating coherent Raman signals were developed, fitting within a housing with an outer diameter of less than 1 mm (Deladurantaye et al. 2014; D. T. DePaoli et al. 2018; Saar et al. 2011). Other flexible nonlinear endoscopic systems utilize photonic crystal fibers to deliver femtosecond pulses, allowing the highly efficient generation of CARS, TPEF, and SHG in biological tissues (Ducourthial et al. 2015; Lombardini et al. 2018). Typically, these devices offer a field of view of up to 450 μm and a lateral resolution of approximately 1 μm. Currently, the research focuses on improving imaging quality (Pikálek et al. 2022).

Rigid endoscopes for nonlinear microscopy employ graded-index (GRIN) lenses. Currently, GRIN lenses can be manufactured with lengths of several centimeters, enabling CARS/TPEF/SHG endoscopic systems for in vivo preclinical studies on nervous tissue (Huland et al. 2012; Zirak et al. 2018). Furthermore, endoscopic THG imaging has been achieved using GRIN lenses (Kuzmin et al. 2016), expanding the range of imaging modalities. The feasibility of an SRS endoscope has been demonstrated in principle (Saar et al. 2011), but further testing on biological tissue is still required.

Multiphoton microscopy and endoscopy use laser excitation with power levels up to a few tens of mW. By using laser beams in the NIR range (750–1000 nm), the risk of photodamage is minimized due to the low absorption of biological tissue at these wavelengths. However, the possibility of inducing photodamage with strongly focused ultrashort pulses cannot be completely ruled out when using laser power suited for high-quality label-free imaging (Bégin et al. 2011; Evans and Xie 2008; Gao et al. 2011; Meyer et al. 2013). The topic of photodamage caused by label-free multiphoton microscopy has been extensively studied by various research groups in different biological systems (Fischer et al. 2008; Fu et al. 2006; Hopt and Neher 2001; Nan et al. 2006; Saytashev et al. 2012; Tinevez et al. 2012) and tissue types (Galli et al. 2014). These studies have shown that high-quality, label-free multiphoton imaging is indeed achievable without photodamage. Additionally, an increase in background fluorescence detected by TPEF may serve as an intrinsic marker for any eventually arising photodamage (Galli et al. 2014).

The specific morphological information and chemical selectivity provided by label-free multiphoton microscopy are well suited for automated image analysis. Each nonlinear signal generates a single-channel intensity image that can be analyzed in various ways, including intensity analysis, area measurement, structure evaluation, form analysis, directional analysis, and other morphological parameters. For instance, automated collagen quantification and fiber orientation analysis based on SHG images have already been established as reliable approaches (Dudenkova et al. 2019). The average orientation and directional anisotropy of myelinated nerve fibers can be evaluated, for example, using 2D Fourier-transform algorithms, which can be useful for assessing myelin health (Bégin et al. 2013). Segmentation algorithms have been developed for evaluating morphological parameters of myelin sheaths (Bégin et al. 2014). Furthermore, software for automated axon segmentation based on CARS images is available (Zaimi et al. 2016) and has been used to create a database of spinal cord structure (Saliani et al. 2017).

## Label-free analysis of nervous tissue in healthy and diseased states

**Fig. 1:**
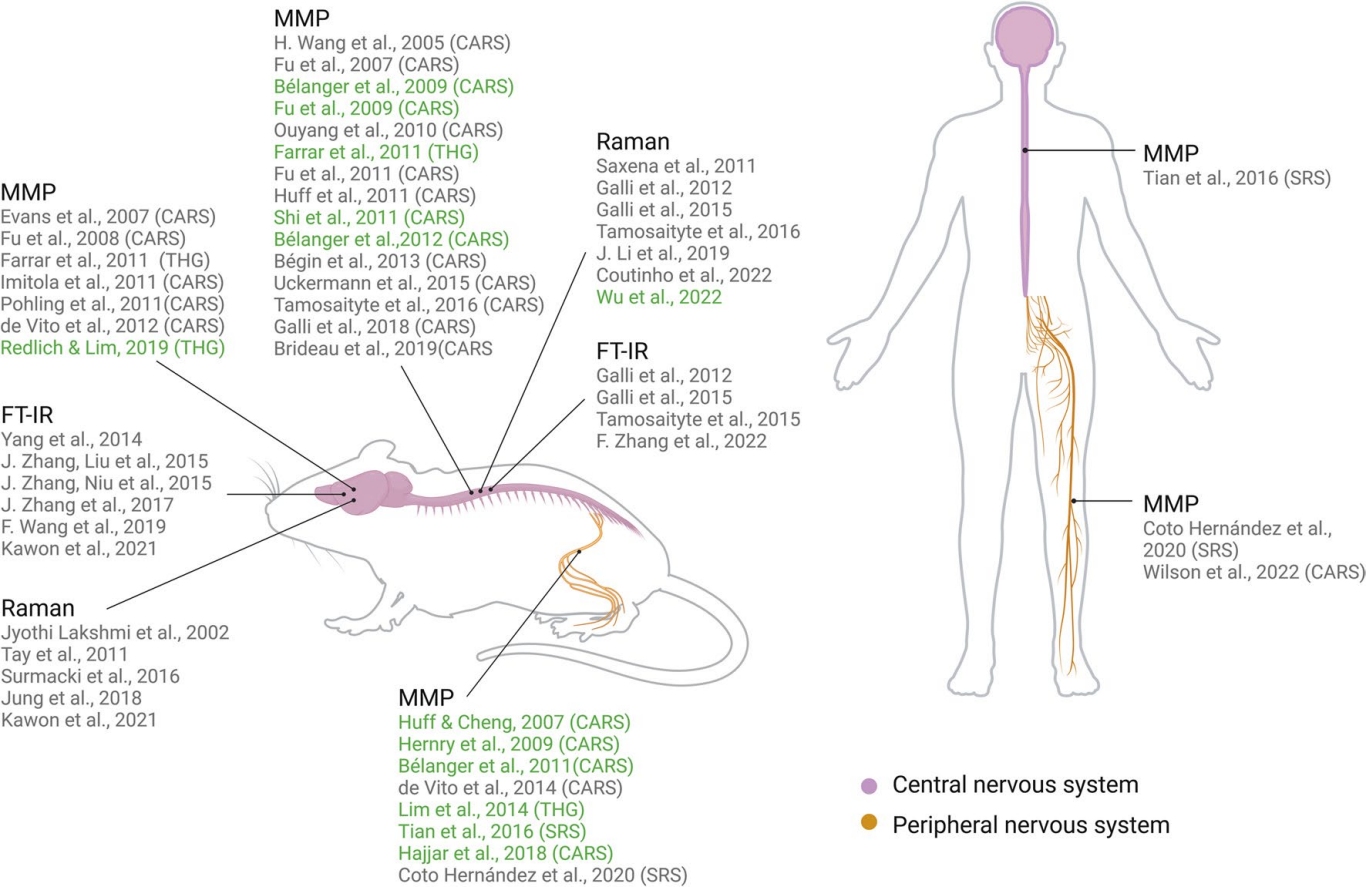
Applications of vibrational (Raman and FT-IR) spectroscopy and label-free multiphoton microscopy techniques to study the central and peripheral nervous systems in rodent models and humans. References in green indicate in vivo studies. In the case of multimodal multiphoton microscopy (MMP), the key technique used for visualization of myelin is reported. Created with BioRender.com

The utilization of optical label-free techniques for imaging and characterizing nervous tissue has a history spanning nearly two decades. Over this time, optical spectroscopy and label-free multiphoton microscopy have been extensively employed in various studies (Fig. 1), showcasing their potential in elucidating the mechanisms of nervous tissue damage and characterizing the effects of experimental therapies.

### Healthy nervous tissue

The CNS of vertebrates comprises several cell types, including neurons, astrocytes, oligodendrocytes, and microglia (resident immune cells). Neurons with myelinated axons transmit stimuli along white matter tracts in the brain and spinal cord. Myelin, a lipid-rich substance produced by oligodendrocytes, forms sheaths surrounding axons and enabling rapid saltatory conduction of action potentials. Thicker myelin sheaths generally result in faster conduction, and the myelin thickness is often quantified by the g-ratio, which represents the ratio of axon diameter to the myelin tube diameter. The morphology of nodes of Ranvier, as well as paranodal and juxtaparanodal regions, also correlates with conduction properties (Suminaite et al. 2019).

In the PNS of vertebrates, myelinated axons are surrounded by collagenous ECM, known as the endoneurium. Axons are further organized into fascicles that are encapsulated by the perineurium. Finally, the epineurium wraps around the entire nerve, providing protection against external stresses. Perineurium and epineurium are also made of collagenous tissue (Griffin et al. 2013).

**Fig. 2:**
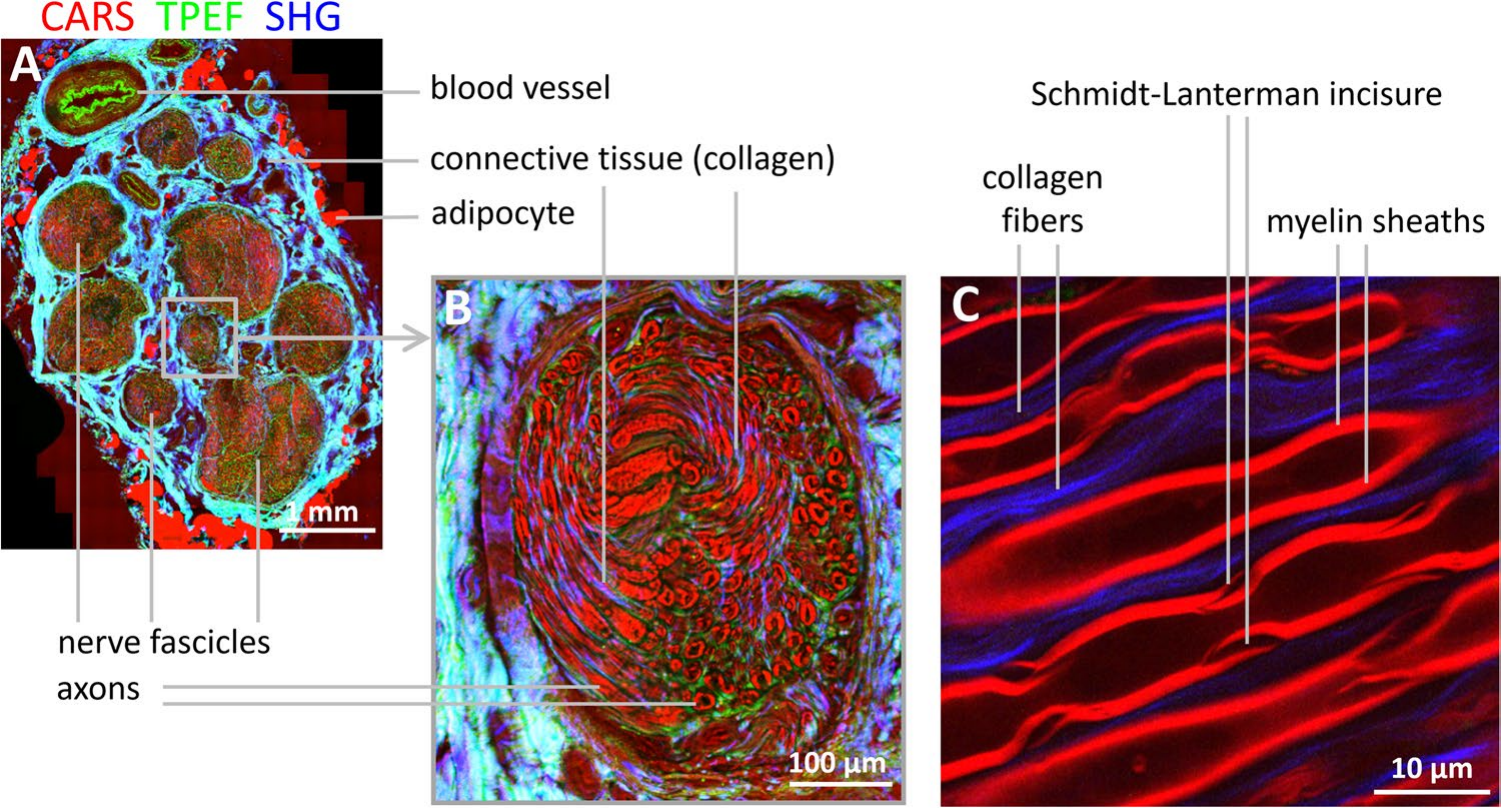
Label-free multimodal multiphoton microscopy of a human peripheral nerve (nervus suralis). The three acquired signals are merged into an RGB image, where CARS is represented in the red channel, TPEF in the green channel, and SHG in the blue channel. **A** Stitched overview image of a transverse section illustrating the macrostructure of the nerve. It shows the nerve fascicles, collagenous connective tissue of the epi- and perineurium, blood vessels of various sizes, and adipocytes surrounding the nerve. **B** Detailed view of a single fascicle, revealing both transversely and longitudinally cut axons, as well as the collagen fibers present between the axons (endoneurium). **C** High-magnification image of a transverse section highlighting the structure of myelin sheaths, including incisures, and the endoneural collagen fibers

Spectroscopy has not been employed for the biochemical characterization of normal nervous tissue. Instead, it has primarily found its application in investigating biochemical alterations associated with injuries or diseases, as discussed in the relevant sections. Conversely, multiphoton microscopy has gained widespread use for examining the micro-morphology of nervous tissue in a label-free manner. Imaging techniques such as CARS allow for the visualization of lipid-rich myelin surrounding axons in spinal cord white matter and peripheral nerves with sufficient spatial resolution. SHG imaging can simultaneously visualize connective tissue and collagen fibers. TPEF imaging provides additional information about cellular and extracellular structures, contributing to the visualization of tissue morphology. Figure 2 exemplifies multimodal multiphoton microscopy of a peripheral nerve, illustrating how different structures can be imaged using these techniques.

### Characterization of healthy nervous tissue by label-free multiphoton microscopy

Label-free multiphoton microscopy has been extensively employed to investigate myelin sheaths in both spinal cords and peripheral nerves of rodent models. The utilization of CARS microscopy for visualizing axonal myelin and elucidating detailed structures such as the node of Ranvier and Schmidt-Lanterman incisures under physiological conditions was first demonstrated by H. Fu et al. (2005). Moreover, the response of myelin to electrical stimulation was investigated in real time using CARS imaging of myelin in spinal tissues, revealing paranodal myelin retraction during electrical stimulation of fresh spinal cord white matter strips, as demonstrated by Huff et al. (2011).

In 2007, in vivo imaging of the mouse sciatic nerve was reported in microscopy by Huff and Cheng using epi-detected CARS and SHG (Huff and Cheng 2007). This study illustrated the possibility of non-invasively imaging physiological myelinated axons and the surrounding collagen fibers following a minimally invasive surgical procedure. This visualization was achieved through CARS imaging for axons and SHG imaging for collagen fibers. In live rats, an experimental design was established to enable CARS imaging at the single axon level, allowing the imaging of myelin vesiculation, macrophage uptake of myelin debris, and spontaneous remyelination over a 3-week period (Shi et al. 2011). To achieve minimally invasive imaging of cellular processes in vivo within the mouse spinal cord, a commercially available micro-endoscopic GRIN lens was combined with a multiphoton microscope. This system enabled simultaneous CARS imaging of myelin sheaths and TPEF imaging of microglia and axons. Morphometric analysis was employed to quantify differences in myelin thickness and microglial motility (Bélanger et al. 2012).

CARS microscopy has proven to be effective in studying brain tissue in animal models (Evans et al. 2007; Fu et al. 2008; Pohling et al. 2011). Fresh unfixed ex vivo mouse brain tissue was successfully imaged using CARS microscopy to identify normal gray and white matter structures on various scales, including myelinated brain fibers. The ability to image myelinated fibers without the need for exogenous labeling allowed for white matter mapping in whole brain slices and analysis of the microstructural anatomy of brain axons. The combination of CARS with TPEF enabled multimodal imaging of myelinated axons and other cells.

Furthermore, the impact of laser beam polarization on CARS contrast in myelin imaging was investigated, revealing how the orientation of the myelin membrane relative to the laser polarization plane affects the morphometric parameters that can be extracted through image analysis, as first demonstrated by Bélanger et al. (2009). Later on, dedicated CARS systems were developed to achieve high-resolution, chemically, and orientationally sensitive imaging of myelinated tissue (Brideau et al. 2019). Additionally, a method based on rotating-polarization CARS imaging, developed by de Vito, was utilized to assess the health status of myelin and initially tested on mouse sciatic nerves (de Vito et al. 2012, 2014). Unlike other techniques, this method exploits the intrinsic molecular architecture of myelin, relying directly on its high degree of molecular order. Using this approach, the spatial anisotropy of CARS was examined, and subtle effects of fixatives on the molecular order of myelin were visualized (de Vito et al. 2020).

SRS imaging has emerged as a sensitive and quantitative tool for studying myelin and nervous diseases in recent years. SRS, in combination with rapid automated quantification of myelinated axons using a machine learning algorithm, enabled histomorphometry of peripheral nerves, as first demonstrated by Coto Hernández et al. (2020).

THG is another label-free nonlinear imaging method that allows for in vivo imaging of myelin in the vertebrate nervous system. For example, THG imaging of the mouse spinal cord enabled simultaneous visualization of myelin sheaths and of individual axons labeled with a fluorescent dye visualized by TPEF, as shown by Farrar et al. (2011). THG microscopy also facilitated label-free imaging of myelinating cells and provided insights into the structure of various myelin domains, including juxtaparanodes, Schmidt-Lanterman incisures, and Cajal bands in the murine brain cortex. Morphometry of myelin, described by the g-ratio, was determined under physiological conditions in mouse models of hypomyelination, and during postnatal development (Lim et al. 2014). By leveraging the deep penetration of IR excitation, THG imaging successfully visualized myelinated fibers in live mouse brains up to a depth of approximately 200 μm. This allowed for the visualization of fibers with different orientations and, in combination with semi-automatic axon tracing based on THG signals, revealed the three-dimensional connectivity of the nervous system (Redlich and Lim 2019).

### Spinal cord injury

Spinal cord injury (SCI) is caused by a primary injury followed by a cascade of degenerative processes known as the secondary injury, which can be temporally classified into acute, sub-acute, and chronic phases. The acute phase immediately follows the trauma and involves vascular damage, ischemia/hypoxia, and subsequent damage to lipids and proteins. Necrotic cell death affects both neurons and glia. Inflammation is initiated through the recruitment of resident microglia and immune cells from the bloodstream. As the injury progresses into the sub-acute phase, additional cellular apoptosis, demyelination of surviving axons, matrix remodeling, and the formation of a glial scar at the injury site are observed. The chronic phase is characterized by ongoing degeneration, including the formation of a cystic cavity, progressive axonal die-back, and maturation of the glial scar (Alizadeh et al. 2019). Inflammation persists throughout the chronic phase and can have both beneficial and detrimental effects following SCI (Miron and Franklin 2014). Macrophages and microglia play a crucial role in the CNS wound healing process by clearing damaged cells and myelin debris (Zhou et al. 2014), as well as by expressing growth-promoting factors (David and Kroner 2011). However, the role of inflammatory cells in SCI is a topic of debate (Loane and Byrnes 2010), and chronic inflammation is generally considered detrimental to regeneration (Ankeny et al. 2006).

SCI triggers the formation of a scar around the injury epicenter, traditionally regarded as a major obstacle to axonal regeneration (Fitch and Silver 2008). Following the injury, astrocytes increase their expression of intermediate filaments and glial fibrillary acidic protein (GFAP), leading to hypertrophy. Reactive astrocytes proliferate and form a mesh-like structure of intermingled filamentous processes that isolate the injury. Within the mature glial scar, activated microglia/macrophages occupy the innermost region of the injury site, while reactive astrocytes reside in the perilesional region, forming a cellular barrier. The persistence of the glial scar throughout the chronic stage is recognized as a significant hindrance to axonal elongation (Cregg et al. 2014). In penetrating injuries where the meninges are compromised, a fibrotic scar is also observed as meningeal fibroblasts infiltrate the lesion. Similarly, in contusive lesions, perivascular fibroblasts migrate to the injury site, leading to the formation of a fibrotic scar (Soderblom et al. 2013).

**Fig. 3:**
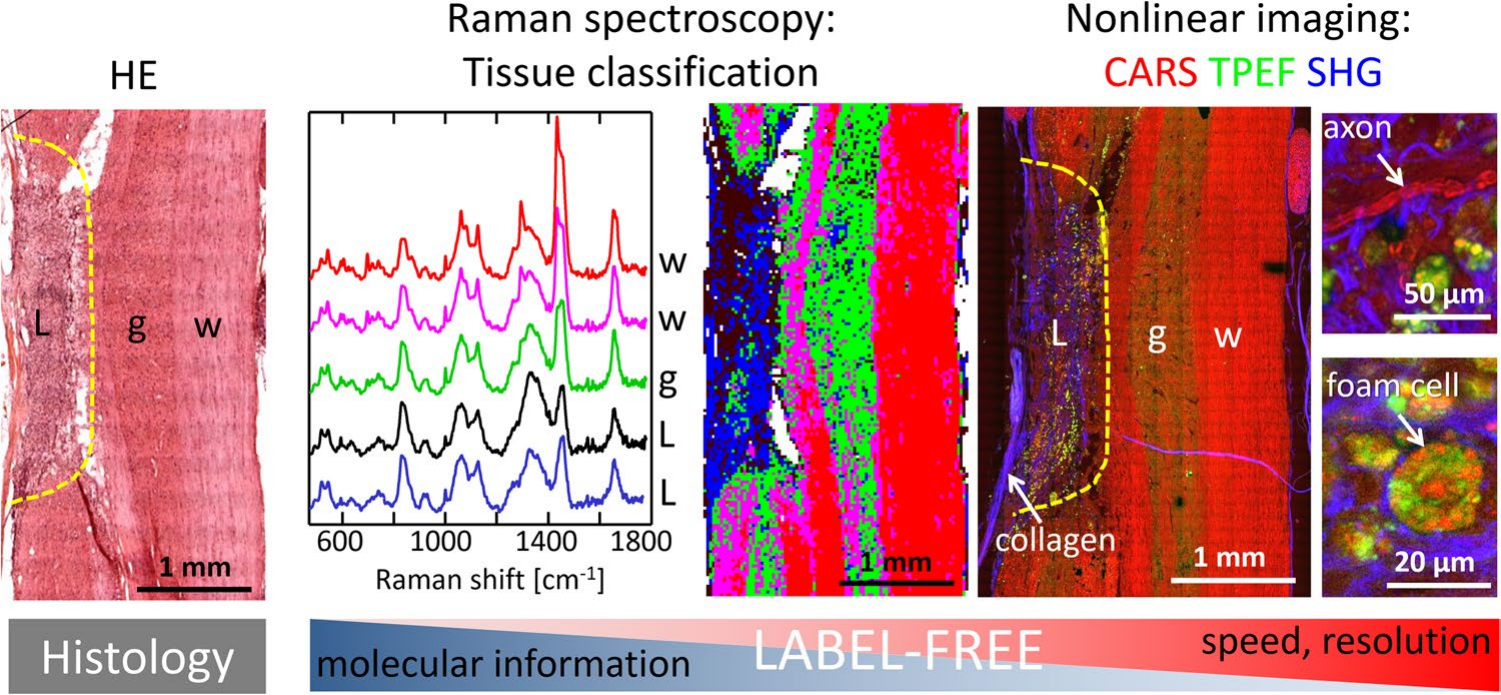
Label-free optical analysis of SCI in a rat model. Raman spectroscopy enables the biochemical characterization of tissue alterations by analyzing changes in spectral bands. Classification of spectral information provides a mapping of tissue regions that correspond to histology (L: lesion, w: white matter, g: gray matter). Multiphoton imaging, achieved through simultaneous acquisition of CARS (red channel), TPEF (green channel), and SHG (blue channel), allows visualization of tissue morphology with sufficient chemical selectivity to distinguish tissue regions and identify additional nervous structures such as axons, inflammatory (foam) cells, and collagen fibers. All images adapted from Galli et al. (2015) and assembled for illustrative purposes

The above-described pathological processes can be investigated using both FT-IR and Raman spectroscopy, as well as label-free multiphoton microscopy. Spectroscopy provides insights into tissue biochemical alterations and enables the identification of lesion-related changes in lipid and protein profiles. On the other hand, multimodal CARS microscopy offers higher lateral resolution and faster acquisition speed while still providing some chemical selectivity (see Fig. 3).

### Characterization of SCI by spectroscopy

In the past, Raman spectroscopy was not frequently utilized for investigating injured nervous tissue. However, there have been notable studies that employed Raman spectroscopy to explore specific aspects of SCI. For instance, Raman spectroscopy was applied to study demyelination and the upregulation of chondroitin sulfate proteoglycans in the extracellular matrix following spinal cord hemisection (Saxena et al. 2011). In a comparative study conducted by our group, we demonstrated the complementary information provided by FT-IR imaging, Raman mapping, and CARS microscopy in addressing demyelination and fibrotic scarring (Galli et al. 2012, 2015). Additionally, a further study confirmed the capability of confocal Raman spectroscopy to identify the characteristic degenerative features of SCI, such as neuron apoptosis, hemorrhage, demyelination, and upregulation of chondroitin sulfate proteoglycans (Li et al. 2019b). Raman spectroscopy has also been employed to retrieve a biochemical signature of lipid metabolism in macrophages and map inflammation within SCI (Tamosaityte et al. 2016). Furthermore, Raman spectroscopy has been used to investigate the biomedical evolution of spinal cord injury and the therapeutic outcomes of low-level laser therapy (F. Zhang et al. 2022), as well as the effects of amniotic membrane treatment in injured rat spinal cords (Coutinho et al. 2022).

### Characterization of SCI by label-free multiphoton microscopy

The morphology of myelin sheath alterations and interactions with nervous and inflammatory cells has been studied using CARS microscopy in various animal models of mechanical and pharmacological spinal cord injury. Sub-micrometric resolution CARS imaging of myelin sheaths in fresh tissues revealed paranodal myelin splitting and retraction following glutamate application in both ex vivo and in vivo guinea pig spinal cords (Fu et al. 2009). Furthermore, the degradation of myelin induced by lysophosphatidylcholine was investigated in ex vivo spinal tissues using CARS imaging, where myelin swelling was monitored by a decrease in CARS intensity and loss of excitation polarization dependence (Fu et al. 2007). Immunofluorescence and CARS imaging techniques were employed to visualize myelin retraction at the nodes of Ranvier and pathological changes in ion channels in white matter strips from guinea pig spinal cords (Ouyang et al. 2010).

The combination of CARS with SHG and TPEF provides unprecedented opportunities for integrated characterization of SCI, allowing visualization of myelin damage together with inflammation and fibrotic scarring simultaneously. Specifically, our group focused on investigating the label-free detection of macrophages and activated microglia in SCI using endogenous TPEF signals. Previous reports have already indicated significant endogenous fluorescence in the cytoplasm of microglia/macrophages (Molcanyi et al. 2013; Oehmichen et al. 1986). Exploiting this endogenous information, we successfully identified activated microglia/macrophages at the single-cell level in the rodent spinal cord by correlating TPEF-positive cells with those identified through immunohistochemistry (Uckermann et al. 2015). Additionally, label-free TPEF imaging was employed to identify activated microglia and track temporal changes in their activity following injury (Galli et al. 2018; Tamosaityte et al. 2016). This information is crucial for future studies investigating inflammation modulation as a therapeutic target. For example, recent studies have shown that pharmacological inhibition of microglia proliferation after SCI in mice and nonhuman primates can lead to improved outcomes, and the combination of CARS microscopy with fluorescent imaging of stained microglia allowed for the direct correlation of improved myelination with reduced microglia proliferation (Poulen et al. 2021).

One of the main limitations of both spectroscopy and label-free multiphoton microscopy techniques is their inability to effectively address the glial scar. To date, no research group has succeeded in identifying a spectroscopic signature specific to the glial scar. In label-free multiphoton microscopy, reactive astrocytes do not exhibit autofluorescence, making it challenging to differentiate them from other structures. Moreover, the spectral resolution of many CARS systems, which use picosecond laser excitation, is insufficient to separate the signals of lipids and proteins. This limitation hinders the clear distinction between elongated axonal structures, astrocytic protein filaments, and collagen fibers that make up the fibrotic scar. However, stimulated Raman scattering (SRS) microscopy shows promise in addressing this gap by enabling the visualization of astrocytic processes through selective chemical imaging of proteins, as demonstrated in human brain tumor samples (Ji et al. 2013). Further investigation is required to determine if glial processes and collagen fibers can be effectively discerned using this technique. A multimodal approach that incorporates SHG imaging may be necessary to achieve this goal.

### Spectroscopy and label-free multiphoton microscopy for studying experimental treatments of SCI

Only a few therapies for SCI are currently employed in clinical settings, primarily focusing on early injury management. These include decompression surgery and treatment approaches aimed at reducing acute damage (Ahuja et al. 2017; J. Wang and Pearse 2015). A few pharmacological neuroprotective treatments have shown promise in preclinical studies and achieved the stage of clinical trials (Badhiwala et al. 2018). Experimental approaches aimed at promoting axonal regeneration are being investigated in preclinical and early clinical trials. These include regenerative strategies involving neural scaffolds for axon guidance, scar inhibition, delivery of therapeutic drugs, and transplantation of cells (Dalamagkas et al. 2018). Therapeutic implants composed of different materials are being extensively studied. Natural materials such as collagen, chitosan, and alginate hydrogels are utilized to construct scaffolds due to their biocompatibility, biodegradability, cellular interaction, and biological functionality. Synthetic polymers are also being explored as scaffold materials in regenerative medicine, offering customizable porosity as well as physicochemical and mechanical properties (Liu et al. 2019). Rats are the preferred animal model for investigating SCI and evaluating the effects of therapeutic strategies in preclinical studies (Metz et al. 2000), benefiting from standardized evaluation of functional outcomes (Basso et al. 1995) and tailored injury models (Cheriyan et al. 2014).

Spectroscopy and multimodal multiphoton microscopy have been employed not only to study degenerative processes after SCI but also to investigate the effects of therapeutic implants. The first study, conducted in 2011, examined the successful repair of traumatically injured rat spinal cord using nanoscale block copolymer micelles. In vivo repetitive imaging in rat models was performed using CARS microscopy to measure the intra- and extra-axonal space in uninjured, compression-injured, and injured/micelle-treated white matter (Shi et al. 2011).

Afterwards, there has been a focus on studying the effects of implants, particularly alginate hydrogels, which offer the advantage of biocompatibility in clinical applications (Anker et al. 2015), as well as ease of handling and modification prior to implantation. The potential therapeutic effect of alginate hydrogels was extensively investigated using rat models of hemisection, including functional assessments. Spectroscopy was initially employed to evaluate the stability of the implants during the chronic injury phase, with FT-IR spectroscopy demonstrating that the implanted material retained its chemical structure over time (Tamosaityte et al. 2015). Spectroscopy and label-free multiphoton microscopy were then utilized to examine the effects of alginate implants and investigate potential correlations between tissue features and functional outcomes. In similar rat models of hemisection, both FT-IR spectroscopy (Tamosaityte et al. 2015) and multiphoton microscopy (Galli et al. 2018) provided consistent findings. These techniques enabled the detection and quantification of improved contralateral demyelination in implanted animals during the chronic injury phase, primarily due to the neuroprotective effects of alginate implants. Additionally, both techniques facilitated the assessment of fibrous scar extension. Multiphoton microscopy, with its superior lateral resolution compared to IR spectroscopy (0.6 μm vs. 2.7 μm), allowed for detailed morphological characterization of the fibrotic scar. SHG imaging resolved collagen fibers, enabling analysis of their orientation and the evaluation of the effects. Furthermore, CARS microscopy enabled the imaging of single-regrowing axons, while TPEF imaging provided insights into the effects of alginate implants on inflammation.

### Traumatic brain injury

Label-free characterization techniques that have been applied to the study of injured nervous tissue and treatment effects in SCI can also be extended to research on traumatic brain injury (TBI), as both conditions share many common features. TBI involves a primary injury, including intracranial hemorrhage and diffuse axonal injury (Mckee and Daneshvar 2015; Su and Bell 2016), as well as a secondary injury characterized by cerebral edema and ischemia (Ghajar 2000). TBI triggers an endogenous and exogenous neuroinflammatory response that exhibits many similarities to SCI (Loane et al. 2014; Morganti-Kossmann et al. 2007). The astroglial response involves significant proliferation and hypertrophy in the lesion penumbra, leading to the formation of a glial scar. Similar to SCI, the astrocytic scar acts as a major barrier to axonal regeneration in the brain (Burda et al. 2016). However, the complex processes underlying brain injury are not yet fully understood, highlighting the need for methods that enable the probing of the molecular signature of injured brain tissue with high spatial resolution (Ercole et al. 2017).

### Spectroscopy in experimental studies of TBI

FT-IR spectroscopy imaging has been successfully employed to map diffuse axonal injury (Wang et al. 2019a; Yang et al. 2014) and investigate its temporal evolution after the initial insult (Zhang et al. 2017). The sensitivity of the Amide I band to protein conformation has been utilized to identify alterations in the protein profile between injured and normal axons in rat models of TBI, potentially serving as a marker for diffuse axonal injury (Zhang et al. 2015a; Zhang et al. 2015b). Besides changes in protein content, also alterations in lipids’ content have been identified by FT-IR spectroscopy in rodent models (Kawon et al. 2021). Raman spectroscopy was also applied to various TBI models, allowing for the detection of hemoglobin presence and alterations in lipid and protein profiles in preclinical pilot studies (Jyothi Lakshmi et al. 2002; Surmacki et al. 2016; Tay et al. 2011). Changes in the Amide I band were observed as well, but their interpretation is more complex compared to FT-IR spectroscopy due to the substantial overlap between lipid and protein signals in Raman spectra. The application of multivariate methods for analyzing Raman spectra may be more effective in evaluating neuronal injury and drug effects, as demonstrated in the study of cerebral ischemic stroke (Jung et al. 2018). While CARS and SRS microscopy have not yet been utilized in the study of TBI, their ability to visualize lipids and proteins may be effectively employed to investigate axonal damage in this context.

### Peripheral nerve injury

Peripheral nerve injury (PNI) can occur as a result of stretch, compression, or laceration (Burnett and Zager 2004). Unlike the spinal cord, peripheral nerves have the ability to repair themselves. However, the restoration of function depends on the severity of the injury. In less severe injuries where the epineurium and perineurium are intact, axons can regrow guided by the mesenchymal nerve structures, leading to a high likelihood of full functional recovery. In more severe injuries where the nerve mesenchymal structures are damaged or the nerve is severed, surgical intervention is necessary for recovery (Beris et al. 2019).

Following PNI, a series of degenerative processes primarily occur in the distal part of the nerve, depending on the severity of the injury. Wallerian degeneration initiates within hours of the injury and primarily affects the distal region. This process involves the swelling and fragmentation of axons and myelin, followed by the disintegration of nerve conductive structures. After the clearance of axonal and myelin debris by Schwann cells and macrophages, nerve regeneration begins a few weeks after the injury and, in most cases, leads to the reinnervation of the organ. In more severe injuries, retraction of the severed nerve fiber ends is observed, and a dense fibrous scar forms at the injury site, hindering the regrowth of axons to reach the distal nerve stump (Wang et al. 2019b). Chronic injuries characterized by scarring and delayed or failed reinnervation remain unresolved problems (López-Cebral et al. 2017). Consequently, various strategies are being investigated to reduce scarring and promote axonal growth after surgical reconnection of the nerve stumps. These strategies include nerve entubulation, graft implantation, and molecular therapies (Carvalho et al. 2019; Duraikannu et al. 2019; Modrak et al. 2020). Rat models are commonly employed for testing new treatments and gaining new insights into degenerative and regenerative nerve processes to identify novel therapeutic targets (Vela et al. 2020).

### Label-free multiphoton microscopy in experimental studies of PNI

In a rat model of sciatic nerve crush injury, myelin changes were monitored using functional assessments and CARS imaging both ex vivo and in vivo for up to 4 weeks after injury to assess the nerve microenvironment (Henry et al. 2009). Histomorphometry of myelinated axons enabled the quantification of demyelination proximal to the crush site (ex vivo) and remyelination distal to the crush site (in vivo) (Bélanger et al. 2011). The evaluation of myelination in injured human nerves has taken the first steps toward translation to human diagnostics using human nerve biopsies (Wilson et al. 2022). SRS microscopy with combined imaging of lipids and proteins displayed the morphology of fascicles and the amount of myelin. These findings may pave the way for intraoperative assessments of the nerve stump’s quality, overcoming the need for frozen section sequences.

Furthermore, label-free multiphoton microscopy has been employed to investigate nerve injury and regeneration in the octopus, which serves as a fully regenerative model (Imperadore et al. 2017). In experimental neuroscience, the omission of markers allows for better comparison of preclinical data across different animal species, as the obtained information is independent of staining protocols specific to each model. For example, multimodal label-free multiphoton microscopy combining CARS, TPEF, and SHG imaging has been used in octopus PNS injury to examine the mechanisms of degeneration processes, scarring and subsequent regeneration of the pallial nerve and of the whole arm (Imperadore et al. 2018, 2022).

### Neurodegeneration

The application of new optical techniques can provide valuable insights into the research on neurodegenerative diseases, particularly multiple sclerosis (MS). MS is an autoimmune disease that affects the brain and spinal cord, leading to inflammatory processes, demyelination, and axonal degeneration. The complex processes involved in MS pathogenesis have not yet been fully elucidated (Lemus et al. 2018; Rodríguez Murúa et al. 2022). Intensive experimental studies have been conducted using animal models, such as experimental autoimmune encephalomyelitis (EAE), which exhibits several features resembling MS, including neuroinflammation, astrocytic reactivity, demyelination, and axonal damage (Constantinescu et al. 2011).

CARS microscopy was used to investigate the processes of demyelination and remyelination in animal models of EAE (Bégin et al. 2013; Fu et al. 2011; Imitola et al. 2011). In our studies, we demonstrated the ability to visualize plaques and associated inflammatory cells in EAE mouse models using label-free CARS and TPEF imaging, respectively (Uckermann et al. 2015). Additionally, a combination of CARS, TPEF, and SHG imaging showed promise in measuring myelin outcomes in a rodent model of Charcot-Marie-Tooth disease, which is a chronic inflammatory demyelinating neuropathy (Hajjar et al. 2018).

SRS microscopy was employed to monitor degeneration in mouse models of amyotrophic lateral sclerosis (ALS) and ALS autopsy materials. Serial imaging enabled long-term observation of disease progression and the effects of drugs in living animals. Very interestingly, three-dimensional imaging of pre-symptomatic mouse models revealed peripheral nerve degeneration coinciding with the earliest signs of muscle denervation, preceding motor function decline (Tian et al. 2016).

## Conclusions and outlook

The utilization of new optical techniques in the field of neuroscience has provided significant advancements in our understanding of various neurological conditions. From spinal cord injury to peripheral nerve injury, traumatic brain injury, and neurodegenerative diseases like multiple sclerosis, these optical techniques have demonstrated their potential in unraveling the complex processes underlying these conditions and evaluating the efficacy of therapeutic interventions.

FT-IR spectroscopy and Raman spectroscopy have proven to be effective in characterizing molecular changes associated with nervous tissue injury, such as axonal damage, protein alterations, and demyelination. These spectroscopic techniques offer valuable information about the molecular signatures of injured tissues, contributing to our understanding of the pathological processes and providing potential markers for diagnostic purposes. Especially Raman spectroscopy is now on the verge of routine application in oncological neurosurgery (D. DePaoli et al. 2020; Galli et al. 2023). Advanced Raman devices and endoscopes designed for medical applications, allowing sterilization protocols, are readily available (Daoust et al. 2021; Desroches et al. 2015, 2019; Lakomkin and Hadjipanayis 2019; Mowbray et al. 2021). Furthermore, the development of disposable Raman probes may further enhance the convenience and accessibility of these diagnostic tools (Shu et al. 2021). Additionally, it is worth noting that confocal Raman spectroscopy can be paired with Brillouin spectroscopy, allowing for the simultaneous investigation of both biochemistry and biomechanics at the subcellular level (Rix et al. 2022). Brillouin imaging spectroscopy has already demonstrated its potential in characterizing the viscoelastic properties of nervous tissue, as evidenced by recent studies (Cheburkanov et al. 2023; Rad et al. 2022; Ryu et al. 2021), while few pioneering applications have addressed the study of neurodegenerative diseases and spinal cord injuries in animal models (Palombo et al. 2018; Schlüßler et al. 2018).

Label-free multiphoton microscopy techniques, including CARS, SRS, TPEF, SHG, and THG microscopy, have emerged as powerful tools for visualizing and assessing neural tissue in a non-invasive and high-resolution manner. These techniques have enabled researchers to investigate the microenvironment of injured nerves, evaluate myelin changes, study neuroinflammatory responses, and monitor disease progression in preclinical models. Exciting new possibilities could come for the development of three-photon excited fluorescence imaging applied to unlabeled tissue, which counts to date only limited applications (Boppart et al. 2019; You et al. 2018). It offers higher penetration depth compared to the above techniques (Xiao et al. 2023), and its endoscopic application on unstained tissue has already been reported using a GRIN lens-based device (Huland et al. 2013).

Lastly, it is important to highlight that within the range of label-free imaging tools available to neuroscientists, there exists a whole family of well-established time-resolved techniques. Notably, time-resolved fluorescence spectroscopy and multiphoton fluorescence lifetime imaging are among them (Marcu and Hartl 2012). While delving into these techniques exceeds the scope of this review, they have the potential to offer specific insights into cell metabolism and neuroinflammation (Cleland et al. 2021; Sagar et al. 2020).

With further advancements and refinement, all these optical methods hold great potential for enhancing our understanding of neurological disorders and facilitating the development of novel therapeutic approaches. Indeed, there are still several technical and medical challenges that need to be addressed in future research on multiphoton imaging of nervous structures. Improving needle-like endoscopic systems and their imaging depth is a critical aspect, particularly in the context of studying deep-seated brain structures or spinal cord injury. From a medical perspective, the translation of these optical techniques to clinical applications remains a key challenge. The development of robust and user-friendly imaging systems, along with standardized protocols for data acquisition and analysis, is crucial for their adoption in clinical settings. The first steps for integration of label-free multiphoton microscopy in medical devices have been done (Li et al. 2019a), but further development is still required. Additionally, validation studies using human tissue samples and clinical trials are necessary to establish the diagnostic and prognostic value of optical imaging in neurological disorders. Furthermore, there is a need to explore the potential of optical techniques for guiding surgical interventions and monitoring treatment responses.

In conclusion, while optical techniques have already contributed to our understanding of nervous tissue injury and repair, further advancements are needed to overcome technical challenges and bridge the gap between research and clinical applications. Addressing these issues will pave the way for the development of novel diagnostic tools, therapeutic strategies, and personalized medicine approaches for nervous systems’ injury, degeneration, and regeneration.

## Data Availability

No new data or materials were presented in this review.
